# Exploratory Analysis of Tannic Acid–Induced Antiproliferative Effects in SH-SY5Y Neuroblastoma Cells: Associations with Toll-like Receptors and microRNAs

**DOI:** 10.3390/biomedicines14051117

**Published:** 2026-05-14

**Authors:** Tuba Gül, Mücahit Seçme

**Affiliations:** 1 Department of Neurology, School of Medicine, Ordu University, 52200 Ordu, Türkiye; tubayazici@odu.edu.tr; 2 Department of Medical Biology, School of Medicine, Ordu University, 52200 Ordu, Türkiye

**Keywords:** tannic acid, neuroblastoma, SH-SY5Y cells, toll-like receptors, microRNAs

## Abstract

**Background/Objectives**: Neuroblastoma is the most frequent solid tumor outside the brain in children and is associated with unfavorable outcomes in high-risk patients. Tannic acid, a naturally occurring polyphenolic compound, has been reported to exhibit anticancer activity; however, its molecular effects in neuroblastoma remain incompletely characterized. The present study aimed to evaluate the antiproliferative effects of tannic acid in SH-SY5Y neuroblastoma cells and to explore its potential associations with Toll-like receptor (TLR) signaling and selected microRNAs in an exploratory and correlative manner. **Methods:** Cell viability was assessed using the CCK-8 assay, which showed that tannic acid was associated with reduced cell proliferation in a dose- and time-dependent manner. Changes in the expression of TLR-related genes and selected microRNAs were analyzed by real-time PCR. TLR-4, NF-κB, and Caspase-3 protein concentrations were determined using ELISA assays. **Results:** Tannic acid treatment was associated with decreased expression of several TLR genes, with statistically significant reductions observed in *TLR2, TLR4*, and *TLR7*. Consistently, protein analyses indicated reduced NF-κB levels. MicroRNA analysis revealed a tendency toward increased expression of hsa-miR-146a-5p, whereas no significant changes were detected in other examined microRNAs. **Conclusions:** Overall, these findings suggest that tannic acid exhibits antiproliferative activity in SH-SY5Y cells and is associated with alterations in TLR-related gene expression and microRNA profiles. However, these observations are descriptive and correlative in nature and do not establish direct mechanistic relationships. Further in vivo and functional studies are required to validate these findings and to clarify their potential biological and therapeutic relevance.

## 1. Introduction

Neuroblastoma is the most frequently diagnosed extracranial malignancy in childhood, arising from neural crest-derived cells [1,2]. It is the most prevalent solid tumor in infancy and is the second most common solid cancer in children and adolescents, following brain tumors. Neuroblastoma is responsible for approximately 15% of cancer-related deaths in the pediatric population [2,3,4]. Neuroblastoma is characterized by significant biological and clinical heterogeneity; therefore, treatment strategies are determined by disease stage, patient age, and underlying biological prognostic indicators [5,6,7,8]. Neuroblastoma is stratified into four distinct risk categories: very low, low, intermediate, and high risk. The assignment of patients to specific risk groups exerts a direct influence on both the therapeutic decision-making process and the clinical outcomes thereof. While cure rates reach approximately 90–95% in low- and intermediate-risk cases, patients with high-risk neuroblastoma (HR-NB) achieve substantially lower cure rates, ranging from 50% to 60% [9,10]. Recent insights into the molecular basis of neuroblastoma have facilitated the emergence of targeted therapeutic strategies designed to selectively block key signaling pathways driving tumor proliferation and progression, thereby enhancing clinical outcomes while reducing treatment-related toxicity [4]. In this context, there is significant potential for the development of novel and effective pharmacological agents for the treatment of neuroblastoma, with a focus on bioactive molecules derived from natural compounds, particularly from plants [11,12].

Tannic acid is a naturally occurring hydrolysable tannin that is classified within the phenolic acid family. It is structurally characterized by a central glucose moiety that is esterified with ten gallic acid residues. It is widely distributed among herbaceous and woody plant species, particularly in nuts, grains, tea, oak, and fruits [13,14,15,16].

Tannins are a diverse group of polyphenolic compounds that are widely distributed in plants, where they are primarily synthesized via the phenylpropanoid pathway [15,17,18]. These substances are predominantly localized in the vacuoles of specialized cells, which are often referred to as tannin cells. In addition, they can also be associated with cell walls and extracellular matrices. In contrast to primary metabolic organelles, such as chloroplasts, tannins do not participate in photosynthesis; rather, they fulfill protective and regulatory functions [17,18,19].

Tannic acid exhibits a variety of unique biological properties, including antimutagenic, antitumor, and antimicrobial activities against bacteria and viruses. In addition to its antioxidant and homeostatic functions, tannic acid effectively eliminates free radicals that are involved in the development of various disorders, including allergies, diabetes, neurodegenerative diseases, and cardiovascular conditions [20,21,22,23,24,25]. Furthermore, there is substantial evidence supporting its anticancer potential [15,16,26]. Tannic acid has been reported to exhibit anti-cancer activity in various types of cancer, including breast [16], lung [27], prostate [28], hepatocellular carcinoma [29], and pancreatic cancer [30] by modulating various cellular mechanisms. At the cellular level, tannins have been observed to interact with proteins, lipids, and polysaccharides, thereby influencing membrane stability, enzyme activity, and signaling pathways [31]. In mammalian systems, including cancer cells, tannins have been reported to modulate multiple intracellular mechanisms such as apoptotic cell death stimulation, cell cycle arrest induction, and suppression of pro-survival signaling pathways. While the effects of these proteins are primarily mediated through intracellular targets, extracellular interactions, including binding to membrane proteins and receptors, have also been described in the scientific literature [16,26,27,28,29,30,31].

Toll-like receptors (TLRs) play a crucial role in mediating innate immune recognition and in regulating key cellular processes, including apoptosis, DNA repair, autophagy, and angiogenesis [29,30,31,32,33,34]. TLRs play a crucial role in innate immunity by sensing pathogen-associated molecular patterns (PAMPs) derived from a wide range of microorganisms. When they engage with their ligands, TLRs recruit specific adaptor proteins that activate transcription factors, such as NF-κB (nuclear factor kappa B) and interferon regulatory factors (IRFs). This shapes the magnitude and quality of the innate immune response [35]. The activation of TLRs in immune and cancer cells can inhibit tumor initiation and progression. However, depending on the context, excessive or insufficient TLR signaling may promote tumor survival and metastasis. Therefore, developing both TLR agonists and antagonists is a promising strategy for cancer immunotherapy [36]. Recent evidence indicates that Toll-like receptor signaling is controlled not only by protein-mediated pathways but also by epigenetic modulators, including microRNAs (miRNAs). These short noncoding RNA molecules are known to control gene expression at the posttranscriptional stage by either suppressing the translation of specific target messenger RNAs (mRNAs) or facilitating their degradation [37,38].

Despite the existence of studies examining the mechanisms of action of tannic acid in various types of cancer, the precise mechanisms through which it acts in neuroblastoma remain to be fully elucidated. The objective of this study is to ascertain the anti-proliferative effect of tannic acid in neuroblastoma cells and to elucidate the mechanism of action through Toll-like receptors and associated microRNAs.

## 2. Materials and Methods

### 2.1. Cell Culture

The human neuroblastoma SH-SY5Y (ATCC^®^ CRL-2266™, Manassas, VA, USA) cell line was maintained in Dulbecco’s Modified Eagle Medium (DMEM; Gibco, Grand Island, NY, USA) supplemented with 10% fetal bovine serum (FBS; Capricorn, Ebsdorfergrund, Germany), 0.1 mM non-essential amino acids and 20 U/mL penicillin, 20 µg/mL streptomycin. Cell expansion and preparation for experimental procedures were performed in accordance with a previously published protocol [39]. For experimental treatments, cells were exposed to increasing concentrations of tannic acid in a manner dependent on both exposure time and dosage. SH-SY5Y cells were cultured under standard conditions and were not subjected to any differentiation protocol. Cells were maintained in their undifferentiated, proliferative state throughout the experiments. Under these conditions, SH-SY5Y cells displayed a typical neuroblast-like morphology with minimal neurite outgrowth. Therefore, observations at 24 and 48 h represent treatment-dependent effects rather than distinct stages of neuronal differentiation.

### 2.2. CCK8 (WST-8/CCK8) Assay

The dose- and time-dependent effects of tannic acid on cell viability were detected using the CCK-8 assay (Cell Counting Kit-8; WST-8; Abcam, Cambridge, UK, Lot No: 1028841-1) in accordance with the manufacturer’s guidelines. Cells were seeded into 96-well plates at a density of 3 × 10^3^ per well and then exposed to concentrations of tannic acid (12.5, 25, 50, 100, and 200 μM) for 24, 48, and 72 h. Tannic acid has been dissolved in ethanol at a concentration below 0.1%. Due to the low concentration (at a concentration of 0.5%), the effect of the solvent did not need to be taken into account. Following the assay protocol, absorbance measurements were obtained at 450 nm using an ELISA microplate reader (Biotek-EPOCH2, Winooski, VT, USA). IC_50_ (half-maximal inhibitory concentration) values were calculated using GraphPad Prism software (version 9.4.1). Specifically, nonlinear regression analysis based on a four-parameter logistic (4 PL) model was applied using GraphPad Prism software. Cell viability percentages were determined based on the formula reported in a previous study [11]. Each experiment was carried out in triplicate (*n* = 3) across three independent biological replicates.

### 2.3. Real-Time PCR Analysis

Total RNA was extracted from both control cells and cells treated with the IC_50_ concentration of tannic acid via TRIzol reagent (Invitrogen, Carlsbad, CA, USA), according to the manufacturer’s instructions. The quality and concentration of the isolated RNA were evaluated with a NanoDrop spectrophotometer (BioSpec-nano, Shimadzu, Kyoto, Japan). For cDNA synthesis, the High Capacity cDNA Synthesis kit containing RNase inhibitor (ABT, Cat. No.: C03-01-20, Ankara, Turkey) was employed. Relative RNA expression levels were determined using the Rotor Gene 6000 Real-Time PCR Thermocycler in combination with WizPure™ qPCR master mix (SYBR, W1711, Seongnam, Republic of Korea). The primer sequences employed for amplifying TLR genes are listed in Table 1 [40,41]. For the analysis of microRNAs (miRNAs), a process known as cDNA synthesis was carried out using the miScript cDNA Synthesis Kit with Poly(A) Polymerase Tailing, a product of Abm (Richmond, BC, Canada). The PCR was carried out under the following cycling conditions: an initial denaturation at 95 °C for 15 min, followed by 40 cycles of 95 °C for 15 s and 60 °C for 1 min. Subsequently, the effects of tannic acid on the expression levels of miRNAs, including miR-21, let7-e, miR-155, and miR-146a, were examined using Real-Time PCR. Fold changes in gene expression were determined using the 2^−ΔΔCT^ method, with GAPDH (for mRNAs) and U6-2 (for miRNAs) serving as the housekeeping gene for normalization. Each experiment was carried out in triplicate (*n* = 3) across three independent biological replicates.

### 2.4. TLR-4 and NF-κB and Caspase-3 ELISA Assay

The concentrations of TLR-4, NF-κB, caspase-3 in control and tannic acid–treated cells were measured using commercially available ELISA kits, following the manufacturers’ protocols (NF-κB kit, BT Lab, Shanghai, China, Cat. No.: E0690Hu; TLR-4 kit, Sunred, Shanghai, China; ‘Human Caspase-3 ELISA Kit, Cat. No. E4804Hu, BT LAB). Control and treated SH-SY5Y neuroblastoma cells were lysed in RIPA buffer (Milipore, Burlington, MA, USA), which was supplemented with a protease inhibitor cocktail. The cell samples were then centrifuged at 12,000× *g* for 15 min at 4 °C. The resulting supernatants were collected and used for the assessment of NF-κB, TLR-4 and caspase-3 levels. Protein concentrations were calculated by comparing the values of the absorbances with those obtained from a predefined standard curve. The results are reported as nanograms per milliliter.

### 2.5. Statistical Analysis

PCR results were analyzed using the ΔΔCt method, with data processing and normalization performed via the GeneGlobe RT-PCR Analysis RT^2^ Profiler PCR Array Data Analysis platform (Qiagen, Hilden, Germany). Statistical analyses were carried out using GraphPad Prism version 9.4.1. Data are expressed as mean ± standard deviation (SD) from at least three independent biological replicates. Comparisons between groups were performed using either an unpaired Student’s *t*-test or one-way ANOVA, followed by Dunnett’s post hoc test when applicable. Results were considered statistically significant when the *p*-value was below 0.05 (ns, *p* > 0.05; * *p* ≤ 0.05). Each experiment was carried out in triplicate (*n* = 3) across three independent biological replicates.

## 3. Results

### 3.1. Cytotoxic Activity of Tannic Acid on SH-SY5Y Cells

**Figure 1:**
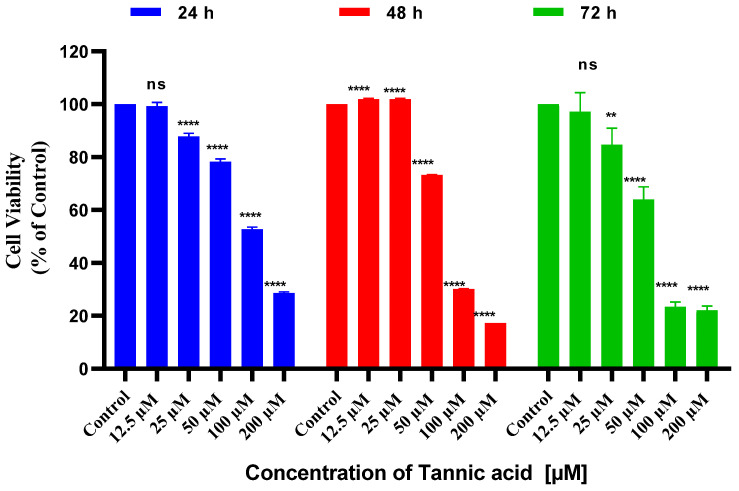
The in vitro cytotoxic effect of tannic acid on SHSY5Y cells after 24, 48 and 72 h. Results are reported as the percentage of normalized cell viability (%) relative to control cells. Differences among group means were evaluated using one-way ANOVA, followed by Dunnett’s post hoc test (ns *p* > 0.05, ** *p* ≤ 0.01, and **** *p* ≤ 0.0001). All experiments were conducted in triplicate (*n* = 3) across three independent biological replicates.

**Figure 2:**
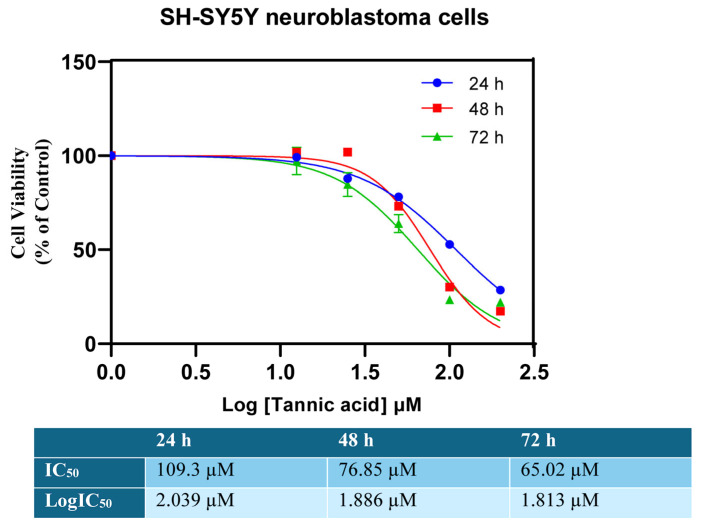
The IC_50_ value of tannic acid was determined to be 109.3 µM at 24 h, 76.85 µM at 48 h and 65.02 µM at 72 h. Results are reported as the percentage of normalized cell viability (%) relative to control cells.

The cytotoxic effect of tannic acid on SHSY5Y neuroblastoma cells was detected using the CCK8 method. The determination of the IC_50_ value was executed by employing the GraphPad program, a computational tool that facilitated the analysis of the 24 h, 48 h, and 72 h results (Figure 1). As demonstrated in Figure 1, tannic acid treatment at concentrations ranging from 12.5 µM to 200 µM exhibited an anti-proliferative effect on SHSY5Y neuroblastoma cells after 24 h. It has been observed that tannic acid exerts a dose-dependent effect on cell proliferation, with this effect remaining constant at high doses. The IC_50_ value of tannic acid in SHSY5Y neuroblastoma cells at 24 h was determined to be 109.3 µM. As illustrated in Figure 2, a graphical representation of the IC_50_ values is provided.

A comprehensive evaluation of the data derived from the 48 h CCK8 assay was conducted, which indicated that tannic acid exhibited a dose-dependent reduction in the proliferation of SHSY5Y cells (see Figure 1 for further details). The IC50 value was determined to be 76.85 µM at 48 h (Figure 2).

Upon examination of the 72 h results, it was noted that they were similar to those observed at 48 h. At 72 h, cell proliferation decreased in a dose-dependent manner, and the IC_50_ value was determined to be 65.02 µM (Figure 1 and Figure 2). The lowest IC_50_ value of 65.02 µM at 72 h was selected as the working dose and used as the dose group in Real-Time PCR and ELISA experiments.

### 3.2. Real-Time PCR Results: Expression Changes in TLR Genes and Related miRNAs

**Figure 3:**
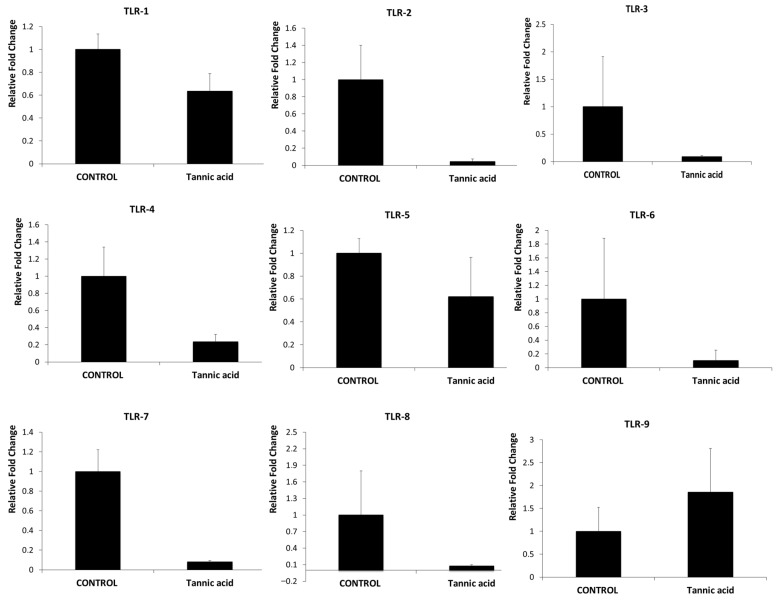
Fold change comparisons in TLR genes between the control group and the group treated with tannic acid. Results were considered statistically significant when the *p*-value was below 0.05. A statistically significant downregulation was observed for *TLR2* (*p* = 0.049987), *TLR4* (*p* = 0.042186), and *TLR7* (*p* = 0.010745). In contrast, the changes observed in *TLR1, TLR3, TLR5, TLR6, TLR8*, and *TLR9* expression levels were not statistically significant (*p* > 0.05).

Changes in the expression of TLR genes and miRNAs associated with this pathway were detected by Real-Time PCR in SH-SY5Y neuroblastoma cells treated with control and tannic acid. Compared to the control, a decrease in mRNA expression was detected in all TLRs except TLR-9 in SH-SY5Y cells treated with tannic acid. Among these decreases, the 23.21-fold decrease in TLR-2, the 4.25-fold decrease in RLR-4, and the 12.47-fold decrease in TLR-7 were found to be statistically significant (*p* < 0.05). The decreases in TLR-1, TLR-3, TLR-5, TLR-6, and TLR-8 expression, which were also identified, were not statistically significant (*p* > 0.05). In addition, tannic acid treatment caused a 1.85-fold increase in TLR-9 expression in neuroblastoma cells compared to the control, but this change was not statistically significant (*p* > 0.05). The fold regulation and *p*-values for these genes are presented in Table 2. The downregulation of Toll-like receptor–related genes in SH-SY5Y cells suggests that the compound may interfere with pro-survival and pro-inflammatory signaling pathways, which have been implicated in tumor progression. Nevertheless, additional functional and in vivo studies about tannic acid are necessary to elucidate the precise function of TLR modulation in the observed anticancer effects. The fold changes in mRNA levels for the genes are shown in Figure 3.

In addition to changes in the expression of toll-like receptor genes, changes in the expression of miRNA-21, let7e, miR-155, and miR146a, which are associated with the TLR pathway, were also investigated by real-time polymerase chain reaction (PCR) following tannic acid treatment. The results obtained demonstrate that only the expression of microRNA-146-5p exhibited a 2.38-fold increase in the group treated with tannic acid. In contrast to the control group, the expression levels of hsa-miR-21-5p, hsa-let-7e-5p, and hsa-miR-155-5p remained unaltered in the tannic acid-treated dose group. However, the investigation did not yield statistically significant results for any of the examined changes in microRNA expression (*p* > 0.05). The change in categories and *p*-values are shown in Table 3.

The results of the present study indicate that tannic acid influences the expression of genes associated with the Toll-like receptor signaling pathway in SH-SY5Y neuroblastoma cells, accompanied by modest alterations in selected microRNA profiles. While the observed downregulation of TLR-related genes and the trend toward increased hsa-miR-146a-5p expression did not reach statistical significance, these findings provide a preliminary framework for exploring the potential molecular mechanisms underlying the biological effects of tannic acid.

The information regarding the target genes of the miRNAs used in the study and their relationship with the Toll-like receptor pathway and the genes involved in this pathway is supported by data from the literature, target identification via the miRDB platform, and pathway relationships derived from the Diana tools-miRPath. The data are also provided in the manuscript supplement (Supplementary Table S1 and Figure S1).

### 3.3. Investigation of the Effects of Tannic Acid on TLR-4, and NF-κB Proteins Using an ELISA Assay

**Figure 4:**
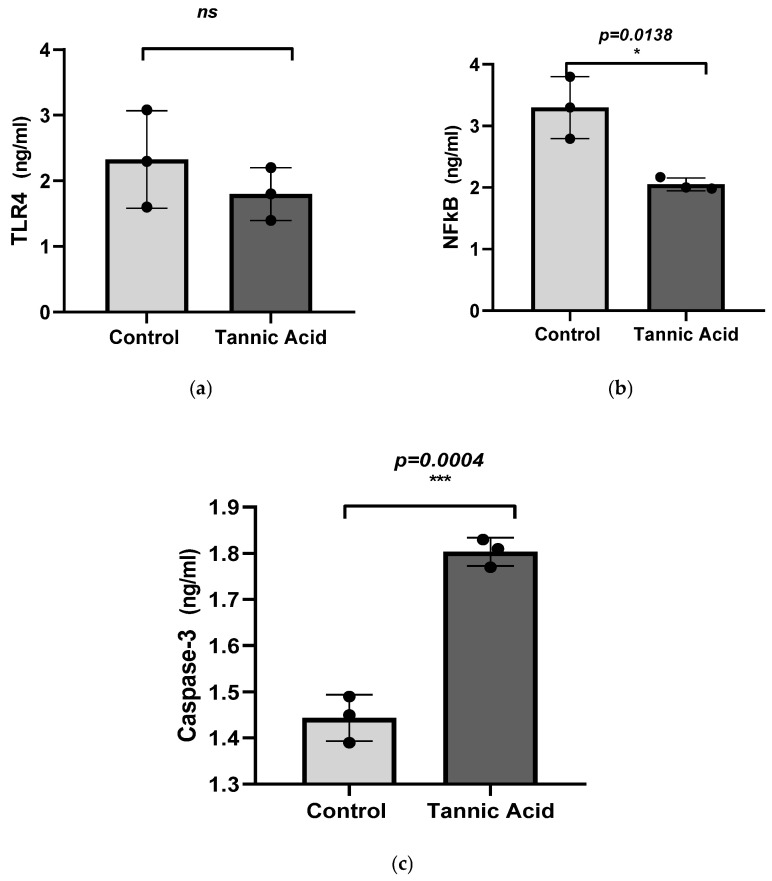
(**a**) Comparison is made between the concentration of TLR4 in neuroblastoma cells treated with tannic acid and the control group, a decrease is observed in the treatment group. However, this decrease was not statistically significant (ns, *p* > 0.05). (**b**) A subsequent comparison of NFkB concentration in neuroblastoma cells within the tannic acid-treated dose group and the control group revealed a decrease in protein concentration. This alteration was found to be statistically significant (* *p* < 0.05). (**c**) It was found that the concentration of caspase-3, a key apoptosis marker, showed a significant increase in neuroblastoma cells treated with tannic acid compared to the control group (*** *p* ≤ 0.001).

The effects of tannic acid on the concentrations of TLR-4, and NF-κB proteins in SH-SY5Y neuroblastoma cells were determined using the ELISA assay. The results obtained indicated that the concentration of TLR4 was 2.32 ng/mL in the control group, while in the dose group treated with tannic acid, this value was 1.8 ng/mL (Figure 4a). These results demonstrate that the concentration of TLR4, a cell survival marker, decreased in neuroblastoma cells following tannic acid treatment. However, this change was not found to be statistically significant (*p* > 0.05). As demonstrated in Figure 4b, the NFkB concentration was ascertained to be 3.29 ng/mL in the control group, whereas this value was determined to be 2.05 ng/mL in the tannic acid-treated dose group (Figure 4b). These results suggest that the concentration of NFkB, a cell survival marker, decreased in neuroblastoma cells following tannic acid treatment. This alteration was investigated to be statistically significant (*p* < 0.05).

Apoptotic activity was assessed by quantifying caspase-3 levels using an ELISA-based method. The results demonstrated that caspase-3 concentration increased from 1.443 ng/mL in control neuroblastoma cells to 1.803 ng/mL in the tannic acid-treated group (Figure 4c), and this increase was found to be statistically significant (*p* < 0.05).

## 4. Discussion

Neuroblastoma is the most prevalent malignancy during infancy and constitutes the most frequent extracranial solid tumor in the pediatric population. Standard therapeutic regimens for high-risk neuroblastoma consist of intensive chemotherapy and surgical tumor removal, followed by high-dose chemotherapy with autologous stem cell transplantation and radiotherapy. Nevertheless, patients with high-risk disease remain highly prone to relapse and frequently experience significant long-term toxicities associated with conventional chemotherapeutic approaches [1,2,3,4,5,12]. Notwithstanding the continuous advancements in therapeutic strategies, the overall survival rate remains below 40%. Consequently, the limited efficacy of surgery and conventional treatment modalities in neuroblastoma—a malignancy arising from the nervous system—has underscored the urgent need for the development of novel and alternative advanced therapeutic approaches targeting neuroblastoma oncogenesis [39]. In this context, research continues on the development of novel agents for all types of cancer, including neuroblastoma [39,40,41,42].

The current study examined the dose- and time-dependent cytotoxic effect of tannic acid on neuroblastoma cells. Tannic acid demonstrated an anti-proliferative effect on neuroblastoma cells, with IC_50_ values determined to be 109.3 µM, 76.85 µM, and 65.02 µM at 24, 48, and 72 h, respectively. It was observed that tannic acid exhibited enhanced efficacy at lower concentrations over time. Tannic acid has been reported to suppress carcinogenesis through several molecular mechanisms, such as modulation of the cell cycle, induction of apoptosis, regulation of cell migration and invasion, and interference with intracellular signaling pathways [26,27,28,29,30]. Tannic acid has been demonstrated to inhibit proliferation in MCF-7, MDA-MB-231, and SKBR3 breast cancer cells, induce G1 arrest in the cell cycle, and trigger apoptosis. Moreover, the study indicated that tannic acid exerts a mechanistic effect by inhibiting EGFR/STAT1/3 and enhancing the p38/STAT1 signaling axis in breast cancer cells [16]. In a recent study, tannic acid was demonstrated to decrease cell proliferation in MCF 7 cells under in vitro conditions, with an IC50 dose of 103.7 μM at 24 h. It has been demonstrated that tannic acid exhibits biological activity by modulating epigenetic mechanisms through the downregulation of the expression of histone deacetylases HDAC1, HDAC2, HDAC3, and HDAC4 [43]. Previous studies have shown that exposure of C4-2, DU145, and PC-3 prostate cancer cells to tannic acid at concentrations between 1.25 and 40 μM significantly reduces cell growth and colony-forming ability in both a concentration- and time-dependent fashion. Moreover, tannic acid has been reported to have no detectable impact on normal PWR-1E prostate epithelial cells. Further evidence indicates that tannic acid promotes apoptotic cell death in prostate cancer cells through modulation of apoptosis-related genes and proteins associated with the endoplasmic reticulum stress–related unfolded protein response pathway [28]. In addition, the results of another study indicated that tannic acid displays cytotoxic activity in HepG2 hepatocellular carcinoma cells. The IC_50_ of tannic acid in HepG2 cells was determined to be 29.4 µM after a 24 h exposure period. In addition, the study demonstrated that tannic acid increases oxidative stress on reactive oxygen species (ROS) and induces apoptosis through caspase activation, thereby exhibiting anti-cancer activity in hepatocellular carcinoma cells [29].

In this study, tannic acid treatment resulted in a general downregulation of Toll-like receptor (TLR) gene expression in SH-SY5Y neuroblastoma cells, with the exception of TLR9. Of particular interest is the statistically significant decrease in TLR2, TLR4, and TLR7 expression, while reductions in other TLRs did not reach statistical significance. In accordance with these transcriptional changes, protein-level analyses revealed a decrease in TLR4 concentration following tannic acid exposure, although this reduction was not statistically significant. Conversely, a substantial decline in NF-κB protein levels was observed, indicating a suppression of downstream pro-survival and pro-inflammatory signaling pathways. The present study demonstrated that the application of tannic acid resulted in an increase in Caspase-3 activity. This finding indicates that the anti-proliferative effect of tannic acid may be initiated by the induction of apoptosis. Collectively, these findings suggest that tannic acid may exert its anticancer effects, at least in part, through modulation of TLR-related signaling pathways. However, further molecular research is necessary to elucidate the biological significance and mechanistic basis of TLR and NF-κB regulation by tannic acid. In consideration of the documented implication of TLR signaling in cancer cell proliferation and survival, the observed repression of TLR-associated gene expression may potentially contribute to the anti-proliferative effects observed in SH-SY5Y cells.

The existing literature on the mechanism of action of tannic acid via Toll-like receptors is limited. A recent study has demonstrated that tannic acid has the capacity to inhibit LPS-induced M1 polarization in macrophages. This inhibitory effect is mediated by direct interaction with TLR4, leading to reduced binding of LPS to the TLR4/MD2 complex and consequent attenuation of downstream signaling pathways [44]. Recent findings suggest that tannic acid exerts its protective role in acute lung injury (ALI) mainly by suppressing TLR4 expression and blocking the activation of ERK and p38 mitogen-activated protein kinase (MAPK) signaling [45]. It has been documented that tannic acid exerts a substantial inhibitory effect on the protein expression of TLR4 and the adaptor protein MyD88 in LPS-stimulated microglial BV2 cells, exhibiting a dose-dependent response. In addition, the anti-inflammatory activity of tannic acid in LPS-stimulated BV2 microglial cells has been shown to be mediated by reducing reactive oxygen species production and inhibiting activation of the NF-κB signaling pathway. A substantial body of research has demonstrated that tannic acid exhibits significant therapeutic potential in the treatment of neurological disorders [46]. It has been demonstrated that chitosan-coated PLGA nanoparticles loaded with tannic acid and vitamin E reduce colon cancer growth via the NF-κB/β-Cat/EMT signaling pathways. In addition, studies have demonstrated that they modulate inflammation through the downregulation of pro-inflammatory cytokines [47].

Downregulation of Toll-like receptor-associated genes in response to tannic acid exposure suggests a potential inhibitory effect on proinflammatory and pro-survival signaling pathways involved in neuroblastoma progression. However, further mechanistic studies are necessary to clarify the functional role of TLR modulation in tannic acid-mediated anticancer activity. In this context, changes in the expression of microRNAs directly related to the toll-like receptor pathway were also investigated. Furthermore, changes in TLR-4, TLR-8, and NF-κB protein concentrations associated with the toll-like receptor pathway in neuroblastoma cells following tannic acid treatment were also investigated using an ELISA assay.

The present study demonstrated that tannic acid treatment led to the downregulation of Toll-like receptor–related gene expression in SH-SY5Y neuroblastoma cells, suggesting a potential modulatory effect on inflammatory signaling pathways implicated in tumor progression. Concurrently, microRNA expression analysis demonstrated that only hsa-miR-146a-5p exhibited a 2.38-fold increase in response to tannic acid treatment, while the expression levels of hsa-miR-21-5p, hsa-let-7e-5p, and hsa-miR-155-5p remained comparable to those observed in the control group. It is noteworthy that none of the detected alterations in microRNA expression reached statistical significance (*p* > 0.05). These findings suggest that while tannic acid may influence TLR-associated gene expression, its effects on the selected microRNAs appear to be limited under the experimental conditions applied.

The scientific findings obtained demonstrate that the TLR signal is also subject to modulation by epigenetic modulators, including non-coding RNAs [37,38]. Recent research has shown that exosomal miR-21 and miR-155 contribute specifically to the crosstalk between neuroblastoma cells and human monocytes in mediating chemotherapy resistance via a novel signaling cascade involving exosomal miR-21/TLR8-NF-κB and exosomal miR-155/TERF1 pathways. Furthermore, evidence has emerged demonstrating that exosomal miR-21 secreted by neuroblastoma cells results in the upregulation of miR-155, which has been associated with TLR8 and NF-κB in human monocytes [48]. In neuroblastoma, it has been demonstrated that the suppression of miR-21 expression results in the conversion of resistant neuroblastoma cells into sensitive cells. Conversely, ectopic expression of pre-miR-21 has been observed to reduce sensitivity to cisplatin treatment. It has been demonstrated that microRNA-21 (miRNA-21) regulates drug resistance and proliferation in neuroblastoma cells, likely by targeting and reducing the expression of the tumor suppressor PTEN [49]. A study reported increased expression of miR-155 and miR-21 in neuroblastoma patients [50]. It has been demonstrated that miR-146a, which has been reported to play a role in the progression and metastasis of various human cancer types due to its abnormal expression, exhibits a potential tumor suppressor gene property by directly targeting BCL11A in neuroblastoma. It has also been reported that miRNA-146a could serve as a novel target for the treatment of human neuroblastoma [51]. Previous studies have reported that miR-146a-5p modulates drug resistance in neuroblastoma by strongly binding to TLR8. It has also been demonstrated that miR-146-5p exhibits strong affinity for TLR8 and is a potent ligand for TLR8 [52]. A study has reported that miR-146a acts as a negative feedback regulator for the NF-κB signaling pathway and also regulates TLR signaling [53]. Tumor-suppressive miRNAs, specifically let-7 and miR-101, have been shown to directly target the MYCN oncogene. This targeting leads to the suppression of cell proliferation and clonogenic growth in MYCN-amplified neuroblastoma cells [54]. In the present study, we examined the alterations in the expression of miRNAs, including miR-21, let7e, miR-21, and miR-155, which have been demonstrated to interact with toll-like receptors in TLR signaling [55], subsequent to tannic acid treatment.

This study has several limitations that should be considered when interpreting the results. First, all experiments were conducted using a single human neuroblastoma cell line (SH-SY5Y). Although this cell line is widely used and well-characterized in neuroblastoma and neurobiology research, the use of a single model system limits the generalizability of the findings, particularly given the known biological heterogeneity of neuroblastoma. Validation of these results in additional neuroblastoma cell lines and in vivo models is necessary to confirm their broader relevance. Second, the concentrations of tannic acid required to achieve anti-proliferative effects (IC_50_ values) are relatively high. This raises important considerations regarding their physiological relevance and translational potential. In vitro studies often require higher concentrations of polyphenolic compounds due to factors such as poor bioavailability, rapid metabolism, and limited cellular uptake. Therefore, the observed effects should be interpreted within the context of experimental conditions, and further studies are needed to evaluate the efficacy of tannic acid at physiologically achievable concentrations. In addition, although protein concentration changes for TLR4, caspase-3 and NF-κB were assessed using ELISA assays, these findings were not validated by complementary techniques such as Western blotting, which represents an important limitation in confirming protein expression changes. While the inclusion of caspase-3 ELISA analysis provides supportive evidence for apoptotic involvement, the study lacks pathway-specific functional experiments to directly elucidate the underlying molecular mechanisms. Taken together, future studies incorporating multiple cell models, in vivo validation, and more comprehensive molecular analyses will be essential to strengthen and extend these findings.

Given the well-established involvement of TLR signaling in inflammatory responses, cell survival, and tumor progression in various cancer models, the observed changes in TLR-related gene expression following tannic acid treatment represent noteworthy molecular associations. However, these findings do not provide direct evidence of interference with pro-tumorigenic signaling pathways. In addition, tannic acid treatment was associated with a 2.38-fold increase in hsa-miR-146a-5p expression, a microRNA reported to be involved in the regulation of TLR and NF-κB signaling. Although the observed increase was not statistically significant, it may indicate a possible trend that merits further exploration. By contrast, the expression of hsa-miR-21-5p, hsa-let-7e-5p, and hsa-miR-155-5p showed no notable changes after treatment. Collectively, these results indicate that tannic acid exposure is associated with alterations in TLR-related gene expression and selected microRNA profiles in SH-SY5Y neuroblastoma cells. However, the present findings should be interpreted as descriptive and correlative. Comprehensive functional and mechanistic studies are required to clarify the biological significance of these molecular changes and to determine their potential relevance in therapeutic contexts.

## 5. Conclusions

In the present study, tannic acid treatment resulted in the downregulation of genes associated with Toll-like receptor signaling in SH-SY5Y neuroblastoma cells. It has been established that aberrant activation of TLR-mediated pathways is associated with enhanced tumor cell survival, proliferation, and resistance to apoptosis in various cancer types. Therefore, the observed suppression of TLR-related gene expression may suggest an interference with pro-tumorigenic inflammatory signaling. While the precise molecular consequences of TLR downregulation were not directly assessed in this study, the findings suggest that tannic acid may exert part of its anti-proliferative effects through modulation of TLR-dependent pathways. Further investigations are warranted to elucidate the downstream signaling events and confirm the biological relevance of TLR suppression in neuroblastoma.

In conclusion, the present findings indicate that tannic acid modulates the expression of TLR signaling components in SH-SY5Y cells, which may contribute to its observed anticancer properties. However, the role of TLR signaling in the comprehensive antitumor mechanism of tannic acid remains to be fully elucidated.

## Figures and Tables

**Table 1:** Reverse and Forward sequences of the primers.

Gene Name	Forward	Reverse
*GAPDH*	GTCTCCTCTGACTTCAACAGCG	ACCACCCTGTTGCTGTAGCCAA
*TLR-1*	CAGCGATGTGTTCGGTTTTCCG	GATGGGCAAAGCATGTGGACCA
*TLR-2*	CTTCACTCAGGAGCAGCAAGCA	ACACCAGTGCTGTCCTGTGACA
*TLR-3*	GCGCTAAAAAGTGAAGAACTGGAT	GCTGGACATTGTTCAGAAAGAGG
*TLR-4*	CCCTGAGGCATTTAGGCAGCTA	AGGTAGAGAGGTGGCTTAGGCT
*TLR-5*	CCTTACAGCGAACCTCATCCAC	TCCACTACAGGAGGAGAAGCGA
*TLR-6*	ACTGACCTTCCTGGATGTGGCA	TGACCTCATCTTCTGGCAGCTC
*TLR-7*	CTTTGGACCTCAGCCACAACCA	CGCAACTGGAAGGCATCTTGTAG
*TLR-8*	ACTCCAGCAGTTTCCTCGTCTC	AAAGCCAGAGGGTAGGTGGGAA
*TLR-9*	GGTTAAAAGACGTTCATCTCCACG	CCTAGCATCCTGAGATACCAGG

**Table 2:** In this study, real-time PCR was used to assess alterations in the expression of genes related to the TLR signaling pathway in SH-SY5Y cells following tannic acid treatment, in comparison with the control group. *GAPDH* was employed as a housekeeping gene. Gene expression in the tannic acid–exposed cells was evaluated relative to controls based on fold change values and associated *p*-values (*: *p* < 0.05).

Gene Symbol	Tannic Acid Dose (IC_50_) Group	
	Fold-Regulation	*p* Value
*TLR1*	−1.58	0.124347
*TLR-2*	−23.21	0.049987 *
*TLR-3*	−11.18	0.228956
*TLR-4*	−4.25	0.042186 *
*TLR-5*	−1.61	0.553926
*TLR-6*	−9.78	0.148987
*TLR-7*	−12.47	0.010745 *
*TLR-8*	−13.36	0.193220
*TLR-9*	1.85	0.269266

**Table 3:** In this study, real-time PCR was employed to investigate changes in the expression of miRNAs related to the TLR signaling pathway in SH-SY5Y cells following tannic acid exposure, in comparison with the control group. *U6-2* was used as the internal reference for normalization. miRNA expression in treated cells was assessed relative to controls based on fold regulation values and the corresponding *p*-values.

miRNA Symbol	Tannic Acid Dose (IC_50_) Group	
	Fold-Regulation	*p* Value
*hsa-miR-21-5p*	1.06	0.973966
*hsa-miR-155-5p*	−1.07	0.774439
*hsa-let7e-5p*	1.14	0.542294
*hsa-miR146a-5p*	2.38	0.087863

## Data Availability

The data sets and analyses from the study can be obtained from the corresponding and co-authors upon reasonable request.

## Supplementary Materials

The following supporting information can be downloaded at https://www.mdpi.com/article/10.3390/biomedicines14051117/s1: Figure S1: Heatmap analysis generated by miRPath for the target pathway analysis of the miRNAs used in the study; Table S1: The information regarding the target genes of the miRNAs used in the study and their relationship with the Toll-like receptor pathway and the genes involved in this pathway is supported by data from the literature, target identification via the miRDB platform.

## Abbreviations

The following abbreviations are used in this manuscript:

NB	Neuroblastoma
TLR	Toll-like receptor
miRNA	Micro-RNA
CCK-8	Cell Counting Kit-8
