# Supplementary material for: Exploratory Analysis of Tannic Acid–Induced Antiproliferative Effects in SH-SY5Y Neuroblastoma Cells: Associations with Toll-like Receptors and microRNAs
Source: Biomedicines. 2026 May 14;14(5):1117. doi: 10.3390/biomedicines14051117 (PMC13204015; doi:10.3390/biomedicines14051117)
Supplement: Supplementary file 1 [file biomedicines-14-01117-s001.zip › biomedicines-4236061-supplementary.pdf]

Table S1. The information regarding the target genes of the miRNAs used in the study and their relationship with the Toll-like receptor pathway and the genes involved in this pathway is supported by data from the literature, target identification via the miRDB platform

Target Detail	Target Rank	Target Score	miRNA Name	Gene Symbol	Gene Description
**https://mirdb.org/cgi-bin/target_detail.cgi?targetID=836473**	386	57	hsa-miR-21-5p	TLR4	toll like receptor 4
**https://mirdb.org/cgi-bin/target_detail.cgi?targetID=412398**	294	76	hsa-miR-155-5p	TLR6	toll like receptor 6
**https://mirdb.org/cgi-bin/target_detail.cgi?targetID=3384453**	573	72	hsa-let-7e-5p	TAB2	TGF-beta activated kinase 1 (MAP3K7) binding protein 2(Toll-like receptor pathway related gene)
**https://mirdb.org/cgi-bin/target_detail.cgi?targetID=46694**	1	100	hsa-miR-146a-5p	TRAF-6	TNF receptor associated factor 6- (Toll-like receptor pathway related gene)
**https://mirdb.org/cgi-bin/target_detail.cgi?targetID=46832**	2	100	hsa-miR-146a-5p	IRAK1	Interleukin 1 receptor associated kinase 1 (Toll-like receptor pathway related gene)

Figure 1.

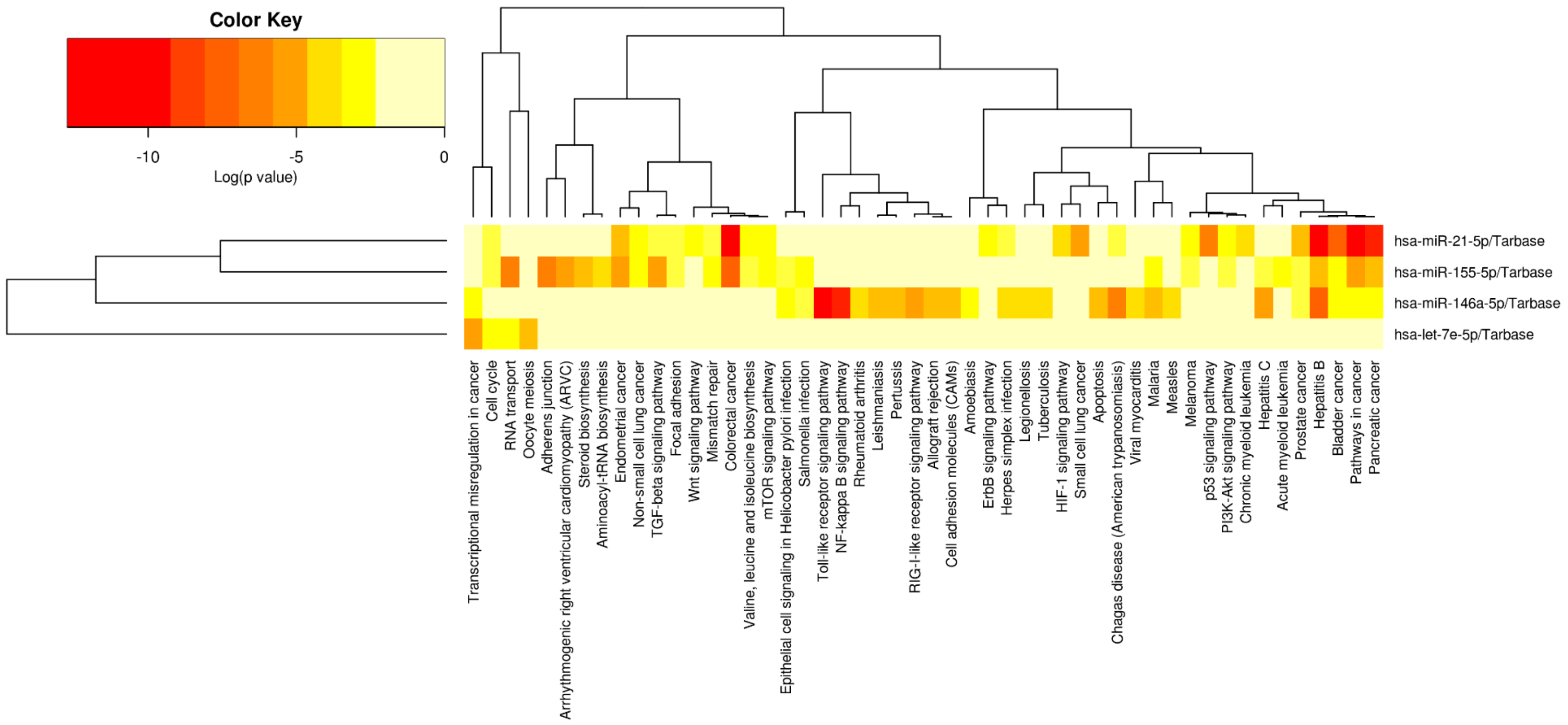

Figure S1. Heatmap analysis generated by miRPath for the target pathway analysis of the miRNAs used in the study
